# Dlc1 interaction with non-muscle myosin heavy chain II-A (Myh9) and Rac1 activation

**DOI:** 10.1242/bio.015859

**Published:** 2016-03-14

**Authors:** Mohammad G. Sabbir, Rachelle Dillon, Michael R. A. Mowat

**Affiliations:** 1 Research Institute of Oncology and Hematology, CancerCare Manitoba, Winnipeg, Manitoba R3E 0V9, Canada; 2 Department of Biochemistry and Medical Genetics, University of Manitoba, Winnipeg, Manitoba, R3E 0J9, Canada

**Keywords:** Dlc1, Non-muscle myosin, Spectrin, Plectin, Rac1, RhoA

## Abstract

The Deleted in liver cancer 1 (Dlc1) gene codes for a Rho GTPase-activating protein that also acts as a tumour suppressor gene. Several studies have consistently found that overexpression leads to excessive cell elongation, cytoskeleton changes and subsequent cell death. However, none of these studies have been able to satisfactorily explain the Dlc1-induced cell morphological phenotypes and the function of the different Dlc1 isoforms. Therefore, we have studied the interacting proteins associated with the three major Dlc1 transcriptional isoforms using a mass spectrometric approach in Dlc1 overexpressing cells. We have found and validated novel interacting partners in constitutive Dlc1-expressing cells. Our study has shown that Dlc1 interacts with non-muscle myosin heavy chain II-A (Myh9), plectin and spectrin proteins in different multiprotein complexes. Overexpression of Dlc1 led to increased phosphorylation of Myh9 protein and activation of Rac1 GTPase. These data support a role for Dlc1 in induced cell elongation morphology and provide some molecular targets for further analysis of this phenotype.

## Abstract (precis)

**Summary:** Dlc1 interacts with several actin filament-associated proteins including Myh9, spectrin and plectin. Transient overexpression of Dlc1 induces an elongated phenotype with elevated Rac1 activation.

## INTRODUCTION

The rat p122^RhoGap^ protein (named DLC1 in humans) was first identified as a RhoA GTPase-activating protein (RhoGap) that binds and enhances the phosphatidylinositol 4,5-bisphosphate (PIP_2_)-hydrolyzing activity of phospholipase C-δ1 (PLC-δ1) (Homma and Emori, 1995; Sekimata et al., 1999). The initial characterisation of p122^RhoGap^ was soon followed by the discovery that the Dlc1 gene was part of a common 600 Kb genomic deletion in primary human hepatocellular carcinomas (HCC) (Yuan et al., 1998). The Dlc1 gene has been found frequently deleted and epigenetically silenced in a variety of human cancers and this has led to the assertion that Dlc1 is a tumour suppressor gene (Durkin et al., 2007; Xue et al., 2008).

The Dlc1 RhoGap protein consists of three structural domains, an amino-terminal SAM2 sterile α motif (SAM2) domain followed by a linker region upstream from the RhoGap domain and a carboxy-terminal StAR related lipid transfer domain (START) (Alpy and Tomasetto, 2005; Durkin et al., 2007). Dlc1 tumour suppressor activity has been attributed to RhoGap dependent (Guan et al., 2008; Kim et al., 2007) and independent activities (Healy et al., 2008; Qian et al., 2007; Wong et al., 2005).

Besides PLC-δ1, Dlc1 has been found to bind many partners that are associated with focal adhesions, caveolae and adherens junctions (AJs) (for review see Braun and Olayioye, 2015; Ko and Ping Yam, 2014). Dlc1 interacts with focal adhesion associated tensin family members (Chan et al., 2009; Liao et al., 2007; Qian et al., 2007; Yam et al., 2006) and its binding to tensin 3 (TSN3) relieves an autoinhibitory interaction between the SAM2 and RhoGap domains (Cao et al., 2012). Dlc1 has been found associated with other focal adhesion proteins talin and focal adhesion kinase (FAK) (Li et al., 2011). Dlc1 is enriched in caveolae and binds caveolin-1 (CAV1) protein (Du et al., 2012; Yam et al., 2006; Yamaga et al., 2004) through Dlc1's START domain (Du et al., 2012). Alpha-catenin, a key component of the adherens junctions, has also been shown to bind Dlc1 and this interaction was important for the stability of AJs (Tripathi et al., 2012).

Two other binding partners of Dlc1 that interact through the SAM2 domain are the multifaceted eukaryotic elongation factor 1A1 (EF1A1) and the PTEN tumour suppressor (Heering et al., 2009; Zhong et al., 2009). The interaction with EF1A1 helps to target it to the cortical actin network and membrane ruffles, where it functions in F-actin bundling and microtubule dynamics to suppress cell migration (Zhong et al., 2009). Knockdown of both Dlc1 and PTEN enhanced cell migration and transwell invasion compared with loss of either gene alone (Heering et al., 2009). Addition of epidermal growth factor to cells results in a reciprocal binding partner switch between TNS3-Dlc1 and PTEN-PI3K into TNS3-PI3K and PTEN-Dlc1 complexes, resulting in localized changes in Rac1 and RhoA activities (Cao et al., 2015).

The role of Dlc1 gene in tumorigenesis is complicated by the presence of multiple transcriptional isoforms, which are expressed under the influence of alternative promoters (Durkin et al., 2002; Sabbir et al., 2010). Three major Dlc1 transcriptional isoforms have been reported in mammals, which are evolutionarily conserved (Durkin et al., 2007; Ko et al., 2010; Low et al., 2011; Sabbir et al., 2010). The Dlc1 isoforms differ mainly in their amino (N) terminal sequences upstream of the SAM2 domain and it has been previously shown that the isoforms are expressed differentially in a tissue specific manner in the mouse (Sabbir et al., 2010).

Previous studies involving Dlc1 used the canonical isoform 2 and the effects of the other Dlc1 isoforms on cytoskeleton and cell morphology are still poorly understood. Therefore, in the present study we have characterized the cell morphological and cytoskeletal changes associated with each isoform transfected into a Dlc1 deficient tumour cell line. In addition, we have identified novel binding partners associated with the various Dlc1 isoforms by mass spectrometry analysis and validated some of these interacting partners. Our study revealed novel interaction of Dlc1 isoforms 2 and 3 with non-muscle myosin heavy chain IIA and B (Myh9, Myh10). Also, Dlc1 transfection was associated with increased Rac1 activation and Myh9 phosphorylation, which may be responsible for some of Dlc1's cytoskeleton changes.

## RESULTS

### Identification and validation of novel interacting protein partners of Dlc1 isoforms

In order to identify and validate novel Dlc1 interacting partners, first we over expressed Dlc1 isoforms in a Dlc1 deficient cell line, OC-033, and identified the interacting proteins by mass spectrometric analysis of the pull down products. Next, we validated the interaction of novel proteins by immunoprecipitation in cells expressing constitutively high levels of Dlc1. Bend3 endothelial and Neu oncogene induced mammary tumour cell lines were used as constitutively high Dlc1 expressing cell types (Fig. S1). Gene trap and reporter based studies previously showed that Dlc1 is highly expressed during embryonic differentiation and in the embryonic and adult endothelial cells of the mouse (Sabbir et al., 2010). A comparison of Dlc1 protein expression between total embryonic tissue and the Bend3 endothelial cell line indicates the presence of the ∼123 kDa Dlc1 isoform 2 (Fig. S1A). The additional bands present above isoform 2 in the embryonic tissue may represent other isoforms (isoforms 1 and 3 have predicted molecular weights of ∼170 kDa and ∼127 kDa, respectively). In addition, the BC-7.2 mouse mammary tumour cell line also appears to express high levels of Dlc1 isoform 2 (∽123 kDa) (Fig. S1B). Therefore, in this study Bend3 and BC-7.2 cell lines have been used for validating the interacting proteins.

**Fig. 1.**
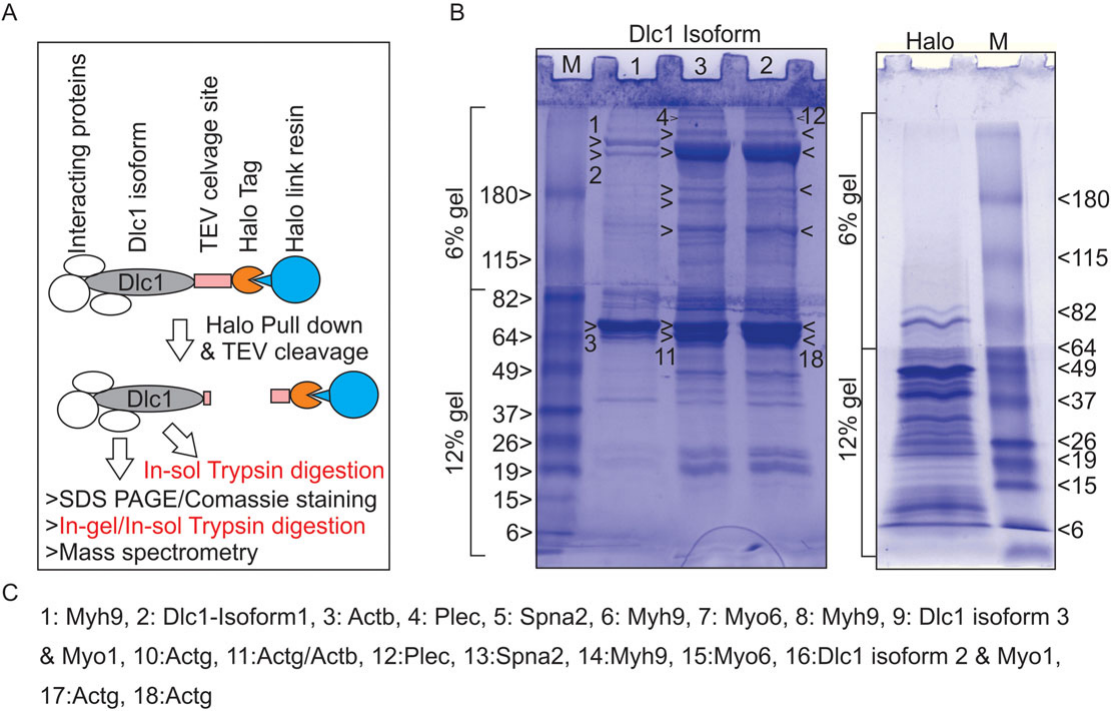
**Identification of interacting partners of Dlc1 protein.** (A) Diagrammatic representation of the Halo-tagged Dlc1 pull-down analysis. (B) Coomassie blue-stained SDS-polyacrylamide gel showing separation of the Dlc1 pull-down products. The excised bands are numbered for in-gel digestion and subsequent mass spectrometry analysis. Only the top and the bottom gel bands have been marked by Arabic numerals due to space constraints. The intervening bands can be counted manually based on the top or bottom number. (C) List of proteins identified by mass spectrometry corresponding to the excised bands shown in the right panel. The proteins correspond to the highest number of peptides found by mass spectrometry.

To understand the functional role of the major Dlc1 isoforms, we wanted to identify the binding partners of the different isoforms. To identify these binding partners, we carried out pull-down experiments using halo-tagged Dlc1 proteins followed by mass spectrometric (MS) analysis (Fig. 1A). The pull-down products were cleaved from the halo-link resin using TEV protease and subjected to in-solution digestion followed by mass spectrometric analysis (Table S1). MS analysis of Dlc1-HaloTag pull-down products included previously identified interacting partners such as caveolin, tensin, 14-3-3, and EF1A along with novel interacting partners (Table S1). In order to eliminate non-specific binding and to identify the most abundant interacting proteins in the Dlc1-HaloTag pull-downs, we further subjected the pull-down products to SDS-PAGE resolution followed by in-gel digestion and MS of the most conspicuous bands (Fig. 1B; Table S2). This approach has led us to identify novel interacting partners including, non-muscle myosin heavy chain 9 (Myh9), plectin (Plec) and alpha spectrin 1 (Sptan1 also known as Spna2 and fodrin) (Fig. 1B,C). These proteins appeared consistently in the pull-down products of all three Dlc1 isoforms however, lower levels of Myh9 were seen in isoform 1 pull-downs compared with isoforms 2 and 3 (Fig. 1B). None of these novel high molecular weight binding partners were seen in the halo-tag-only pull-down experiments (Fig. 1B). In addition, the low molecular weight proteins that were found in the ‘halo control’ sample (Fig. 1B) were not prominently visible in the halo-Dlc1 isoform pull-down samples. There are several explanations which may account for this observation. One possibility is that Dlc1 may have masked the non-specific binding on the halo protein in the halo-Dlc1 fusion protein. Another possibility is that the high molecular weight fibrillar cytoskeletal proteins associated with halo-Dlc1 multiprotein complexes (MPC) may have limited the access to non-specific binding on the halo protein. Moreover, the halo-Dlc1 pull-down fraction was washed stringently to remove non-specific binding which may in turn have eliminated some of the non-specific halo binding proteins. The high stoichiometry of actin and Myh9 binding in the pull-downs suggest that the Dlc1 isoforms 2 and 3 were associating with filamentous actin and Myh9 prior to SDS-PAGE.

**Fig. 2.**
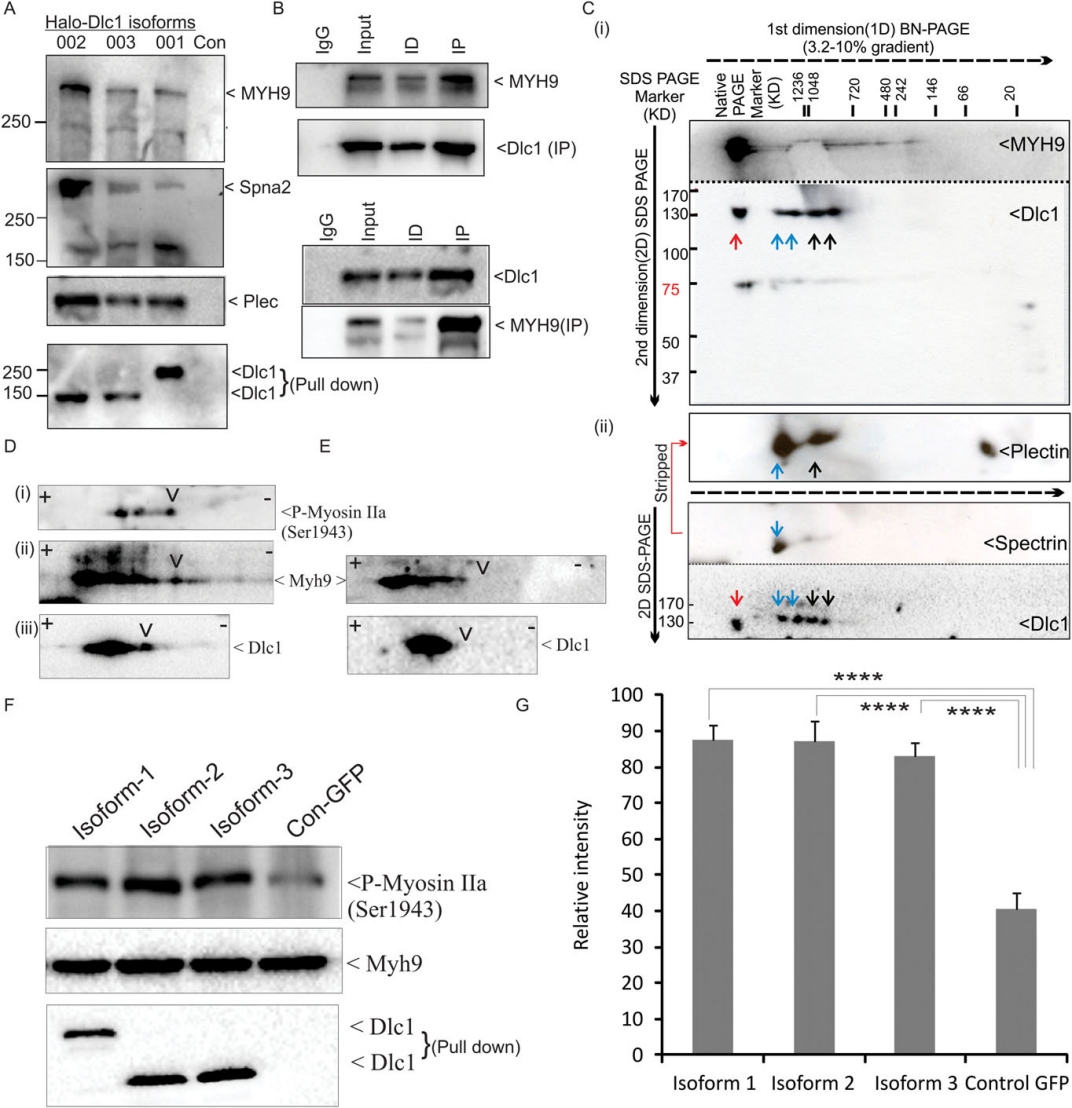
**Validation of Dlc1-interacting proteins by co-immunoprecipitation and BN-PAGE.** (A) Immunoblot blot shows pull-down of Myh9, Spna2 and Plec along with the three Halo-tagged Dlc1 isoforms transiently transfected in OC-033 cells. Eluted extracts from Halo-resin pull-downs were run on SDS-PAGE and blotted with the indicated antibodies. (B) Reciprocal co-immunoprecipitations of Dlc1 and Myh9 in Bend3 cells. ID-immuno-depleted and IP-immunoprecipitated fractions. (C) Immunoblot after 2D BN-PAGE/SDS-PAGE showing multiprotein complexes containing Dlc1. The cellular lysate from BC 7-2 cells was subjected to 2D BN-PAGE/SDS-PAGE and subsequently immunoblotted with anti-Dlc1, Myh9, Plec and Spectrin antibodies. Samples were run in multiple lanes of a single native gel (dotted black line indicates direction of gel movement) and then individual lanes were dissected and run in 2nd dimension SDS-PAGE (solid black arrow indicates direction of gel movement). (Ci) The top panel is a single blot which was cut into two pieces along the dotted line and immunoblotted separately with anti-Dlc1 and Myh9 antibodies and subsequently imaged together. (Cii) The bottom panel is another blot, cut into two pieces and immunoblotted as described above. The top two panels are stripped membrane (red line showing stripping sequence), the bottom one was first immunoblotted with anti-Spectrin antibody and then stripped and immunoblotted with anti-Plectin antibody and subsequently aligned to show relative position. In 2-D BN-PAGE/SDS-PAGE, the interacting monomeric proteins appear on a vertical line. The MPCs involving different interacting partners are marked by red (Dlc1+Myh9), blue (Dlc1+Plec+Spectrin) and black (Dlc1+Plec) arrowheads. (D) Association of Dlc1 with phosphorylated Myh9 (Ser1943**)**: Halo-taged-Dlc1 pull-down product was cleaved by TEV protease, focussed on a pH 3-10 IEF strip and subsequently resolved by 2D-SDS-PAGE. The left panel immunoblot (Dii) indicates that Dlc1 is associated with multiple post-translationally modified forms of Myh9 (black arrowheads), specifically Myh9 phosphorylated at residue Ser-1943 (Di). (E) The immunoblots were obtained following phosphatase treatment of the halo pull-down products. Arrowheads indicate the position of missing phosphorylated bands. (F) Immunoblots indicate relative proportion of p-Myh9 (Ser1943) in cells overexpressing the different Dlc1 isoforms. (G) Bar diagram showing relative quantification of p-Myh9 (Ser1943) in cells overexpressing the Dlc1 isoforms. Data are expressed as the mean±s.d. of 4 independent experiments. ****P<0.001 as calculated by Mann–Whitney test.

Validation of the novel interacting partners of Dlc1 in halo-tag pull-downs was carried out by immunoblotting using specific antibodies against Myh9, Plec and Spna2 proteins in OC-033 cells (Fig. 2A) and BC-7-2 cell lines (data not shown). The Myh9, Plec and Spna2 proteins consistently appeared in the immunoblots using pull-down products, which corroborate the findings in our mass spectrometric analysis (Fig. 2A). In addition, the reciprocal co-immunoprecipitation of Dlc1 and Myh9 in the Bend3 cell line indicates that this interaction is ubiquitous in Dlc1 expressing cells (Fig. 2B).

### Dlc1 multiprotein complexes involve different interacting partners

In order to study the MPC involving Dlc1 and the interacting proteins, we subjected the cell lysates from native Dlc1 expressing cells to blue native polyacrylamide gel electrophoresis (BN-PAGE)-2D-SDS-PAGE analysis (Fig. 2C). BN-PAGE allows separation of multi-protein complexes in their native form and in the 2nd dimension, the interacting monomeric proteins will migrate in a hyperbolic diagonal and the components of one concrete MPC will be found below the diagonal, located on a vertical line. The BN-PAGE analysis revealed that Dlc1 is present in several high molecular weight multiprotein complexes involving Myh9, Plec and spectrin (Fig. 2C). It is interesting to note that the MPC involving Myh9 was not associated with Plec or spectrin (Fig. 2C).

### Dlc1 interacts with phosphorylated Myh9 (Ser-1943)

Recent studies have suggested that phosphorylated Myh9 plays a key role in cell migration (Betapudi et al., 2011; Breckenridge et al., 2009; Dulyaninova et al., 2007). In our mass spectrometric analysis, we observed the presence of several phosphorylated Myh9 peptides, including the S-1943 residue (GPM10000000165). In order to identify if Dlc1 is associated with phosphorylated Myh9, we subjected the Halo-Dlc1 pull-down product to isoelectric focusing followed by 2D-SDS-PAGE and immunoblotting using anti-Myh9 and anti-phospho-Myh9(Ser-1943) antibodies. Immunoblotting revealed that Dlc1 was associated with phospho-Myh9(Ser-1943) (Fig. 2D). Further, phosphatase treatment of the halo pull-down product eliminated the phosphorylated fraction from both Dlc1 and Myh9 confirming the spots observed in 2D-IEF (Fig. 2E). Moreover, we have observed that transient expression of Dlc1 significantly increased the phosphorylated-Myh9(Ser-1943) level in cells (Fig. 2F-G).

### Dlc1 interaction with Myh9 *in vivo*

**Fig. 3.**
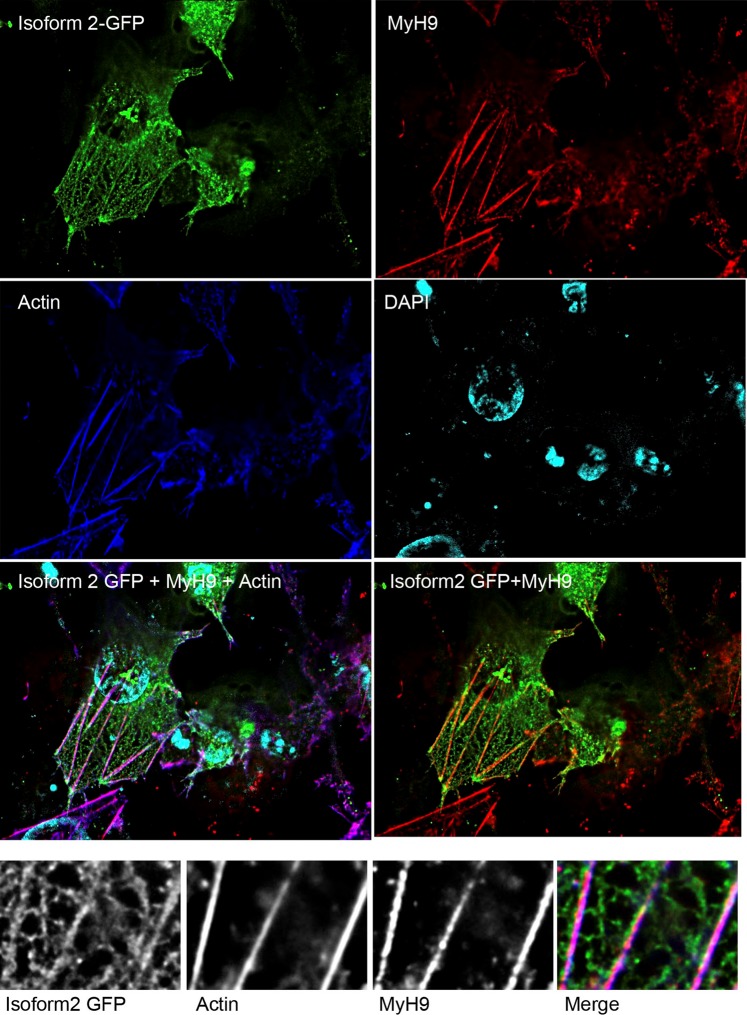
**Co-localization of Dlc1 with Myh9.** Immunostaining of GFP-Dlc1 isoform 2-transfected OC-033 cell line. The cells were stained with an anti-Myh9 antibody, followed by fluorescently labelled secondary antibody and counterstained with Phalloidin (Actin) and DAPI. The actin and Myosin 9 were found to be co-localized with GFP-Dlc1 isoform 2 along the length of the stress fibres. The merged image at the bottom row has the same colour scheme as in the top panels.

Interaction of Dlc1 isoform 2 with Myh9 was further confirmed by immunofluorescence showing the co-localization of GFP-tagged Dlc1 isoform 2 with Myh9 along the length of actin stress fibres (Fig. 3). The association of Dlc1 with stress fibres has been seen to be restricted to 2-4 h of transient expression, over expression gradually leads to complete disappearance of the stress fibres. Also, the GFP-Dlc1 isoform 2 was found associated with a network of filamentous structures cross-connecting the actin bundles in a mesh (Fig. 3).

### Dlc1 over expression induced cell morphological changes

**Fig. 4.**
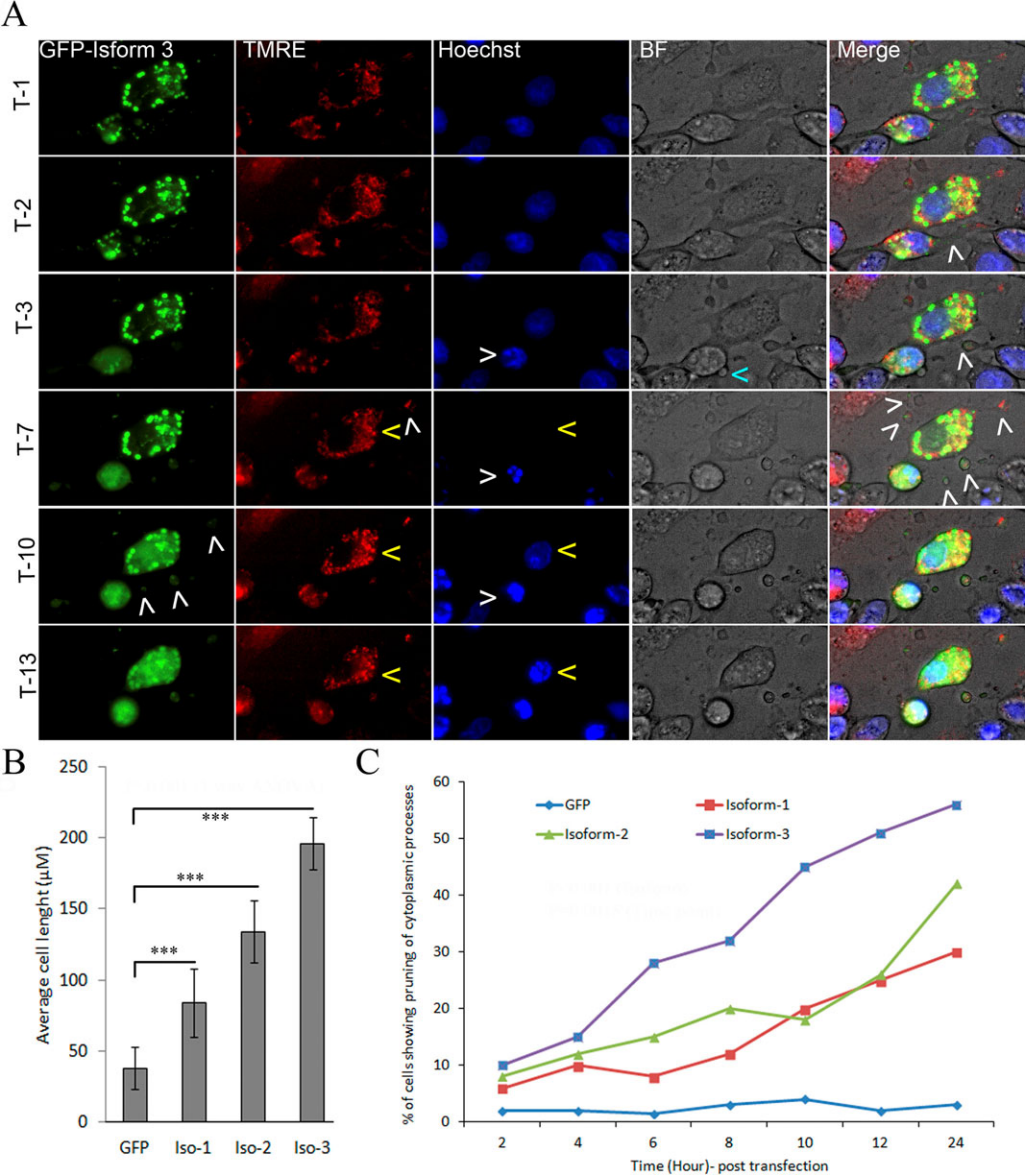
**Effect of Dlc1 isoforms on cell morphology.** (A) Time lapse (every 5 min) images of cells starting at 12 h post transfection with GFP-Dlc1 isoform 3 and stained with tetramethylrhodamine (TMRM) dye. Transfected cells showing extensive cell elongation as well as the tearing off of cytoplasmic processes (white arrowhead on green and merge channels). TMRM and Hoechst dye were used to visualize the integrity of the mitochondria and nucleus, respectively (yellow arrowheads). Apoptotic cells showing reduction of TMRM dye due to loss of mitochondrial membrane potential. T=time unit or time point following 12 h post transfection, each time unit is 5 min in duration. (B) Bar diagram showing average cell length in the transfected cells (mean±s.e.m.). ***P<0.0001 by Mann–Whitney test; when means were compared by one-way ANOVA, *P*<0.001. (C) The percentage of cells showing extended cytoplasmic processes (>150 µm) over time after transfection of the Dlc1 isoforms. Approximately 400 cells were counted in three independent experiments and the results are represented as the average percent. The mean percentage of cells showing extended cytoplasmic processes was compared at different time points between the GFP-tagged isoforms and the control GFP transfection by two-way ANOVA test. The two-way ANOVA test result indicates that the percentage of cells showing extended cytoplasm process at different time points is highly significant (*P*=0.0015). It also indicates that there is a significant difference between isoform 3 and isoforms 1 and 2 (*P*<0.001).

Transient expression of the Dlc1 isoforms for 6-24 h caused significant cell spreading, elongation, and breaking off of cytoplasmic protrusions or pruning in OC-033 cells (Fig. 4; Movies 1-3, Figs S3-S4). Also, prolonged expression of Dlc1 induced the formation of protein plaques inside the cell (Movies 1-3; Fig. 4A). The extent of cell elongation and protrusions was significantly higher (*P*<0.001) with isoform 3 when compared with the other isoforms and showed cytoplasmic protrusions and the formation of profuse filamentous actin rich filopodia all over the surface of the cell (Fig. S2). The fraction of transfected cells showing cellular protrusions was significantly increased over time with all three isoforms (*P*=0.0015), however, isoform 3 showed a significantly higher percentage of pruning over other isoforms at different time points (Fig. 4C). The comparatively higher degree of increase in cell length observed for isoform 3 correlates with the increased cytoplasm pruning. In addition, live cell imaging has shown that elongated cytoplasmic processes in Dlc1 over-expressing cells often tear off from the cell body into isolated cytoplasmic masses leading to loss of cell volume (Fig. 4; Movies 1-3, Figs S3-S4).

Prolonged transient over expression of Dlc1 was lethal in our experiments. In live cell imaging experiments, we have observed that nuclear transport of the Dlc1 isoforms occurs prior to cell rounding, which preceded cell death (Fig. 4; Figs S3-S4). In addition, live cell imaging also revealed that nuclear fragmentation initiated prior to the loss of mitochondrial membrane potential (Fig. 4).

### Dlc1 over expression significantly reduced active RhoA but increased active Rac1

**Fig. 5.**
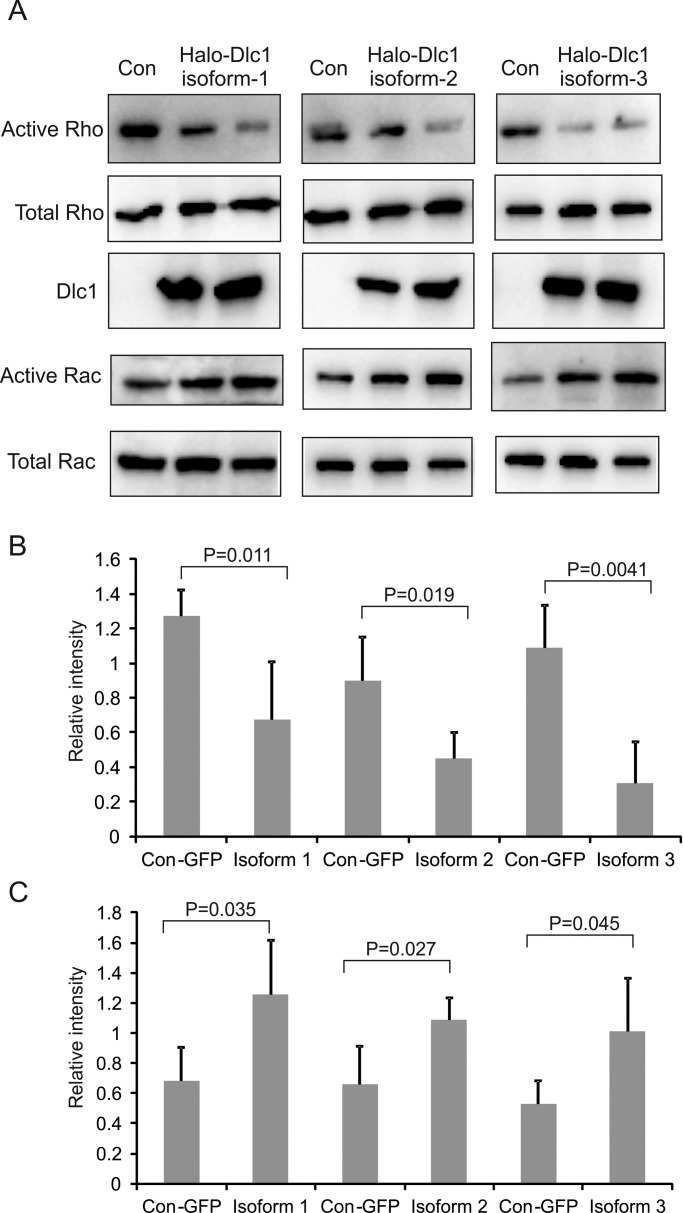
**Active RhoA/Rac1 measurements.** (A) Immunoblot of active (upper) and total (lower) RhoA and Rac1 in Dlc1 isoform-transfected cells as determined by the pull-down assay. (B,C) Histograms showing the ratio of active to total RhoA (B) and Rac1 (C) as determined by densitometry. Data are expressed as the mean±s.d. (*n*=6). *P*-values were calculated using the Mann–Whitney test.

The rat p122^RhoGap^ has been shown to have strong GTPase activity against RhoA but not against Rac1 *in vitro*. Recently, it has been shown that inhibition of Myh9 by deletion or through blebbistatin treatment (an inhibitor of Myh9) activates Akt via Rac1 and PAK1 in gastrointestinal epithelial stem cells (Zhao et al., 2015). In order to see if over expression of Dlc1 affects Rac1 activity, we measured the active RhoA and Rac1 using active Rho, Rac/Cdc42 pull-down assays in the Dlc1 over expressing cells. Interestingly, we have seen that over expression of Dlc1 isoforms 1, 2 and 3 significantly reduced active RhoA (*P* values 0.011, 0.019, and 0.0041, respectively) but increased active Rac1 (*P* values 0.035, 0.027, and 0.045, respectively, Fig. 5).

## DISCUSSION

In this paper we have shown that all three Dlc1 isoforms bind to various actin filament-associated proteins including non-muscle myosin heavy chains, plectin and spectrin. We do not think these associations are due to trapping during cell lysis for three reasons. First, we found Dlc1 in high molecular weight complexes with Myh9, spectrin and plectin after BN-PAGE. Their association in reciprocal immunoprecipitations, and their co-localization by immunofluorescence microscopy in whole cells, support this conclusion. All three Dlc1 isoforms were found associated with these proteins at various levels, with isoform 1 showing reduced association with actin and Myh9 compared with isoforms 2 and 3.

The association of Dlc1 with Plec may explain some of plectin's signalling control downstream of focal adhesions. Plec is a large cytolinker protein that plays an important role in anchoring intermediate filaments (IF) to focal adhesions, desmosomes, the nuclear envelope and cytoplasmic organelles (for review see Wiche and Winter, 2011). Interestingly, plectin-deficient (plectin−/−) fibroblasts showed increases in the number of focal adhesions (FAs) and stress fibre formation suggesting that plectin also plays a regulatory role in actin assembly (Andrä et al., 1998). Plectin binds actin and this interaction is influenced by PIP2 (ibid.). Knockout of plectin results in decreased focal adhesion kinase (FAK) activity and increased levels of active RhoA (Gregor et al., 2014). Loss of Dlc1 results in increased focal adhesions and stress fibre formation (Holeiter et al., 2008; Kawai et al., 2009; Sabbir et al., 2010; Wong et al., 2008). This suggests that plectin's negative control of RhoA and stress fibre formation may be partially mediated through its association with Dlc1, which would turn off RhoA through its RhoGap activity.

Dlc1's association with Spna2 (also known as fodrin) may allow regulation of membrane cytoskeleton dynamics. The membrane cytoskeleton consists of α and β spectrin tetramers linked to short filamentous actin–adducin-4.1 junctional complexes which are anchored to the inner plasma membrane by ankyrin protein complexes (for review see by Baines, 2010). α-Adducin has F-actin capping activity that promotes spectrin binding to actin (Gardner and Bennett, 1987; Kuhlman et al., 1996; Li et al., 1998). Rho associated kinase, ROCK, phosphorylates α-adducin, which in turn promotes spectrin association with F-actin. This association is necessary for the increased membrane ruffling and cell motility seen after TPA and HGF treatment (Fukata et al., 1999). Also, the unphosphorylated form of α-adducin is enriched at cell-cell contacts (Fukata et al., 1999). Therefore, Dlc1's close association with α-spectrin would allow local dampening of ROCK kinase activity resulting in decreased phosphorylation of α-adducin.

It has been known for a long time that non-muscle myosins (NM) IIA and IIB can be activated through Rho signalling (Vicente-Manzanares et al., 2009). Myosin regulatory light chain can be phosphorylated by the Rho effectors ROCK and citron kinase, resulting in activated myosin filament formation, reviewed in (Matsumura, 2005; Zhao and Manser, 2005). As well, ROCK inhibitory phosphorylation of MSB subunit of myosin phosphatase sustains the activation reaction (for review see Ito et al., 2004). The close association of Dlc1 with Myh9 and 10 would be expected to dampen or prevent NM-IIA and IIB filament formation through inhibition of Rho activity.

In recent years, it has come to be appreciated that phosphorylation of NMII heavy chains plays an important role in controlling myosin assembly (for review see Dulyaninova and Bresnick, 2013). We found that Myh9 phosphorylated at serine1943 was enriched in complexes with Dlc1. As well, cells transiently expressing all three Dlc1 isoforms showed increased phosphorylation of Myh9 at position S1943 compared with vector only transfected cells. Phosphorylation at position S1943 by casein kinase II (CKII) *in vitro* is involved in Myh9 filament turnover (Dulyaninova et al., 2007). This association of Dlc1 with S1943 phosphorylated Myh9, suggests that Dlc1 may be involved in reduced Myh9 filament stability. At the same time, Dlc1 would inhibit stress fibre formation through downregulation of Rho and ROCK kinase preventing Myh9 activation through MLC20 phosphorylation.

How elevated Dlc1 expression alters S1943 phosphorylation is unknown at this time. The kinase that phosphorylates Myh9 (IIA) at S1943 *in vivo* is unknown in cells, since knockdown of CKII does not affect phosphorylation of Myh9 (Betapudi et al., 2011). Myh10 (NMHC-IIB) has been shown to be phosphorylated by atypical protein kinase C (PKC) zeta (aPKCζ), which in turn is phosphorylated by and part of a complex with p21-activated kinase 1 (PAK1), a downstream effector of activated Rac1 (Even-Faitelson and Ravid, 2006; Even-Faitelson et al., 2005). However, Myh9 is not phosphorylated by aPKCζ (ibid.). In Dlc1 over expressing cells, it would be expected that increased phosphorylation of Myh10 would be seen due to Rac1 activation. Whether other pathways downstream of Rac1 alter Myh9 phosphorylation needs further study.

It has been known for a long time that Rho and Rac signalling are antagonistic to one another (reviewed in Burridge and Wennerberg, 2004). The mechanism for Rac1 activation in Dlc1 overexpressing cells is presently unknown. A possible mechanism could be through the inactivation of the Filamin A-associated RhoGAP (FilGAP), and the closely related ARHGAP22a (RhoGAP2) RacGAPs, which are activated by ROCK kinase (Ohta et al., 2006; Sanz-Moreno et al., 2008). The reduced Rho and ROCK activation in Dlc1 overexpressing cells would in turn allow local Rac1 activation due to lower FilGAP activity. Another possibility is the ability of Dlc1 to bind PTEN after Erk signalling, which in turn prevents PTEN's ability to inhibit Rac1 activation (Cao et al., 2015). Further experiments will be needed to test these possibilities.

## MATERIALS AND METHODS

### Cell culture

In this study, we have used a Dlc1 deficient ovarian tumour cell line (OC-033) for transfection experiments, which was derived from a heterozygous gene-trapped Dlc1 and K-RasG12D mouse tumour (Dlc1^gt/wt^/LSL-KRas^G12D/wt^) (Fig. S1) (Sabbir et al., 2012). In addition, we have also used a Neu (ErbB2) oncogene-transformed mammary cancer (BC-7-2) cell line, which constitutively expressed high levels Dlc1 protein (Fig. S1) (Dillon et al., 2009). Furthermore, we have used Bend3 endothelial cells as a constitutive native Dlc1-expressing cell line (Fig. S1). All cells were grown in DMEM supplemented with 10% foetal calf serum.

### Plasmids

The full length Dlc1 isoforms, namely isoform 1 (GenBank: HM008381.1), isoform 2 (GenBank: AF178078.1) and isoform 3 (GenBank: AK147539.1) were amplified using different isoform specific PCR primers as follows: isoform 1-cDNA- F, 5′ATGTCTGTAGCTATCAGAAAGAGGAGCTGGGAAG; isoform 2-cDNA- F, 5′CTGCGCCGACCTTAATGTGTAG; isoform 3-cDNA-F, 5′GGTGGATGGGGGACCCCGAGGGC and isoform 1/2/3-cDNA-R, 5′GTTGCAGTCACGGGTGCTTC. The amplified cDNAs were subsequently cloned in the both pEGFP-C1/N1 (Clontech, Mountain View, CA) and pHTN/C HaloTag (Promega, Madison, WI) plasmids. The plasmids containing full-length Dlc1 isoforms were sequenced to verify the lack of any PCR induced mutations and compared with the Genbank sequence.

### Dlc1-HaloTag mammalian pull-down and characterization of protein complexes by mass spectrometry

To identify the protein interacting partners, Dlc1 isoforms were expressed in the OC-033 cell line as both C and N terminal Halo tag fusion proteins and used for the pull-down experiments using Halolink resin (Promega Madison, WI). Briefly, the cell pellet was lysed using Mammalian Lysis Buffer (Cat# G9381, Promega,) and sonicated. The bait-prey complexes containing the Dlc1-Halo tagged fusion protein (bait) and the potential binding partners (prey) were pulled down using HaloLink resin and extensively washed in buffer containing 100 mM Tris (pH 7.6), 150 mM NaCl, 1 mg/ml BSA, 0.05% IGEPAL^®^ CA-630 (octylphenoxypolyethoxyethanol, Cat# I3021, Sigma-Aldrich, Oakville, ON). Finally, the purified bait-prey protein complexes were subjected to overnight digestion with TEV protease at 4°C to release the halo linked Dlc1 protein and the tag free protein complexes were eluted using a His-Trap-Spin column. The eluted protein complexes were subjected to in-solution trypsin digestion followed by tandem mass spectrometry (MS) analysis using AB SCIEX TripleTOF™ 5600 System (Applied Biosystems/MDS Sciex, Foster City, CA) at the Manitoba Centre for Proteomics and Systems Biology. In addition, the purified bait-prey protein complexes were also subjected to SDS-PAGE and stained with colloidal Coomassie stain (Dyballa and Metzger, 2009). The protein bands were excised and in-gel tryptic digestion was performed followed by tandem mass spectrometry analysis using AB SCIEX TripleTOF™ 5600 System (Shevchenko et al., 2007).

### BN-PAGE and second dimension SDS-PAGE

BN-PAGE was performed as described (Fiala et al., 2011). The total cellular lysate was separated on a native gel consisting of 3.2% stacking gel and 4-15% gradient resolving gel in the first dimension until the Coomassie blue reached the bottom of the gel. The gel was then cut into individual strips and denatured in SDS sample buffer (Tris 12.5 mM pH 6.8, SDS 4%, glycerol 20%, Bromophenol blue 0.02%) with or without 1% β-mercaptoethanol. The individual strips were then placed onto 4% acrylamide glycine stacking gel of the 2-D SDS PAGE gel and electrophoresed (Fig. 1C). Proteins were transferred onto polyvinylidene difluoride (PVDF) membrane and immunoblotted with specific antibodies.

### Immunoprecipitation and western blotting

Total cellular proteins were extracted as described (Sabbir et al., 2010). The cell lysate was pre-cleared and immunoprecipitated using anti-Dlc1 and anti-Myh9 antibodies as described (Sabbir et al., 2012). The western blots were immunoblotted with anti-GFP (ab-290), anti-Myh9 (Sc-98978, Santa Cruz Biotechnology (SCBT) Dallas, TX; ARP48072_P050, Aviva Systems Biology, San Diego, CA), anti-phospho-Myh9-Ser-1943 (CST-5026, New England Biolabs, Ipswich, MA), anti-Plectin (sc-33649, SCBT) and anti-Spectrin, antibodies (Sc-32931,SCBT) and visualized. Immuno-blots were scanned using a Storm 840 PhosphorImager scanner and quantified by densitometry using ImageQuant software (version 1.2; both from Molecular Dynamics, Sunnyvale, CA).

### Fluorescence microscopy and live cell imaging

The tumour cells were grown on cover slips as well as glass bottomed cell culture dishes. The cells were transiently transfected with various pEGFP/Halo-tagged Dlc1 isoforms plasmids with Lipofectamine™ 2000 (Invitrogen, Burlington, ON). For immunofluorescence, the cells were fixed with 2% paraformaldehyde for 10 min after 6-24 h after transfection, permeabilized with 0.2% (v/v) Triton X-100 for 10 min at room temperature, and then visualized directly or stained with either TRITC-phalloidin (Sigma-Aldrich, Oakville, ON) for F-actin, and anti-Myh-9 antibodies, followed by incubation with appropriate fluorescent conjugated secondary antibody. The Dlc1 isoforms were also transiently expressed as Halo-tagged fusion proteins in OC-033 cells and live cell imaging was performed using VivaView FL Incubator Fluorescence Microscope (Olympus) at 37°C and 5% CO_2_. Time lapse images were taken at 10 min interval for 48 h using live cell permeable HaloTag TMRDirect Ligand (Promega, Madison, WI) at 5 µM concentration. The microscopic images were processed using public domain software ImageJA 1.45b (http://imageja.sourceforge.net/).

### Active Rho, Rac1 pull-down assay

Active RhoA and Rac1 were analysed using the Rho/Rac/Cdc42-GTP pull-down assay in cells transiently transfected with the Dlc1 isoforms (Ren and Schwartz, 2000). The GST-Rhotekin-Rho binding domain peptide was immobilized on glutathione-sepharose beads (Thermo Fisher, ON, Canada) as described previously (Sabbir et al., 2010). For the active Cdc42/Rac-1 pull-down assay, a GST-fusion of the PAK1 70-117 peptide (Addgene plasmid #12217) was expressed in BL21 cells and immobilized on glutathione-sepharose beads in cell lysis buffer containing 25 mM HEPES (pH 7.5), 150 mM NaCl, 1% Nonidet P-40, 10 mM MgCl2, 5% glycerol, and 1× complete protease inhibitor mixture (Roche, ON, Canada) (Shin et al., 2013). The Dlc1 isoforms expressing cell lysates were sonicated on ice and centrifuged to eliminate the cell debris. Approximately 500 µg of the cell lysate was clarified for nonspecific binding by incubating with 10 µl of GST glutathione-sepharose beads for 1 h at 4°C. The lysate was centrifuged and the supernatant was transferred to a fresh tube containing 50 µg GST-RBD/PAK1 glutathione-sepharose beads and incubated with shaking for 1 h at 4°C. The beads were centrifuged and washed thrice in lysis buffer and the bound proteins were eluted in 50 mM Tris with 1% SDS. The eluted proteins were resolved on 12% SDS/PAGE and immunodetected with anti-RhoA (Cat. #2117, Cell Signal Technology, Danvers, MA) and anti-Rac1 (Cat. #ARC03, Cytoskeleton Inc., Denver, CO) antibodies.
